# Repeatability of endocrine traits and dominance rank in female guinea pigs

**DOI:** 10.1186/s12983-021-00449-2

**Published:** 2022-01-14

**Authors:** Taylor L. Rystrom, Romy C. Prawitt, S. Helene Richter, Norbert Sachser, Sylvia Kaiser

**Affiliations:** 1 Department of Behavioural Biology, University of Münster, Badestr. 13, 48149 Münster, Germany; 2 Münster Graduate School of Evolution, University of Münster, Hüfferstr. 1a, 48149 Münster, Germany

**Keywords:** Glucocorticoids, Endocrine phenotype, Individual variation, Social environment, Stress reactivity, Social rank, Rodent, Variance decomposition

## Abstract

**Background:**

Glucocorticoids (e.g. cortisol) are associated with variation in social behavior, and previous studies have linked baseline as well as challenge-induced glucocorticoid concentrations to dominance status. It is known that cortisol response to an acute challenge is repeatable and correlates to social behavior in males of many mammal species. However, it is unclear whether these patterns are also consistent for females. The aim of this study was to investigate whether baseline and response cortisol concentrations are repeatable in female guinea pigs (*Cavia aperea* f. *porcellus*) and whether dominance rank is stable and correlated to baseline cortisol concentration and/or cortisol responsiveness.

**Results:**

Our results show that cortisol responsiveness (after 1 h: R = 0.635, 95% CI = 0.229, 0.927; after 2 h: R = 0.764, 95% CI = 0.433, 0.951) and dominance rank (R = 0.709, 95% CI = 0.316, 0.935) of females were significantly repeatable after six weeks but not correlated. Baseline cortisol was not repeatable (R = 0, 95% CI = 0, 0.690) and also did not correlate to dominance rank. Furthermore, the difference in repeatability estimates of baseline and response values was due to high within-individual variance of baseline cortisol concentration; the amount of between-individual variance was similar for baseline cortisol and the two measures of cortisol responsiveness.

**Conclusions:**

Females occupying different dominance ranks did not have long-term differences in cortisol concentrations, and cortisol responsiveness does not seem to be significantly involved in the maintenance of dominance rank. Overall, this study reveals the remarkable stability of cortisol responsiveness and dominance rank in a female rodent, and it remains an open question whether the magnitude of cortisol responsiveness is adaptive in social contexts for females.

**Supplementary Information:**

The online version contains supplementary material available at 10.1186/s12983-021-00449-2.

## Background

Social interactions with conspecifics are an important aspect of the environment for group-living animals and often lead to the formation of dominance hierarchies. Dominance hierarchies can benefit group members by enhancing social stability and reducing conflict over limited space and resources [1, 2 p. 273], but fitness benefits can differ among individuals as a result of their status within the hierarchy [3–6]. Female dominance displays are typically less conspicuous than male dominance displays and thus are not as well-studied in group-living mammals [4, 7]. Surprisingly little is known about female dominance hierarchies in non-cooperatively breeding rodents (but see [8–12]).

Glucocorticoids (e.g. cortisol) have been proposed as an endocrine mechanism important for dominance rank acquisition and maintenance [13]. Glucocorticoids are secreted through the activation of the hypothalamic–pituitary–adrenal (HPA) axis and mobilize the energy necessary for appropriate behavioral responses to challenges [14, 15]. Some studies indicate that cortisol responsiveness to an acute challenge could be a potential endocrine mechanism triggering levels of aggressive behavior, whereby higher cortisol responsiveness triggers aggression necessary for high dominance status [16–20]. However, selection line studies in rainbow trout (*Oncorhynchus mykiss*) and laboratory CD-1 mice suggest that individuals selected over generations for lower cortisol responsiveness become dominant [17, 19]. Baseline glucocorticoids have also been linked to dominance rank, although this relationship varies among species and seems to be dictated by the behavioral strategies employed to acquire and maintain dominance [13, 21]. Dominant individuals likely have higher baseline glucocorticoid concentrations if it is energetically demanding to be dominant (i.e. high aggression required to acquire and maintain dominance status; many challenges by subordinates), but if high dominance rank is inherited and/or not routinely challenged, dominant individuals should not have high glucocorticoid concentrations [21]. Meanwhile, if there is an energetic cost to being subordinate such as being subjected to frequent threats from dominant individuals, baseline glucocorticoid concentrations of subdominant individuals are likely to be high [21]. However, coping mechanisms such as social support can lower baseline glucocorticoid concentrations [21, 22]. Therefore, the interaction between glucocorticoids and dominance rank is complex and species-specific, and this interaction may be different for baseline and response glucocorticoid concentrations.

When investigating between-individual variation in multiple traits, it is important to establish whether the traits are stable within individuals, especially if these traits are measured at different timepoints [23]. Indeed, many different behavioral traits are stable across time and context [24]. Historically, female behavior has been dismissed as less stable due to the influence of reproductive state [25], although recent studies have shown that female behavior is at least as stable as male behavior [26, 27]. Recent work has also demonstrated that hormonal patterns can be temporally stable [28–31]. Specifically, testosterone and cortisol responsiveness are repeatable, but results are inconclusive on the repeatability of baseline cortisol. Baseline glucocorticoid levels fluctuate throughout the day with current activity, and meta-analyses have indicated that baseline cortisol is less repeatable than cortisol responsiveness [28, 30, 31]. Repeatability is calculated as the proportion of total phenotypic variance (between- and within-individual variance) that can be attributed to the between-individual component. Therefore, measurements such as baseline and response cortisol concentrations can differ in repeatability due to differences in within-individual variation, between-individual variation, or a combination of both components. Decomposing variance into between- and within-individual components can therefore offer a more detailed explanation for why repeatability estimates differ [32].

This study aims to investigate whether dominance rank, baseline cortisol level, and cortisol responsiveness (here defined as the absolute cortisol concentration after 1 and 2 h of exposure to a stressor) [29, 33, 34] are repeatable and whether dominance rank correlates to cortisol concentrations in female guinea pigs (*Cavia aperea* f. *porcellus*). Guinea pigs are social mammals, and females form linear dominance hierarchies [12]. Female dominance hierarchies in wild and captive groups of wild cavies (*Cavia aperea*) follow an age-graded structure [35], with young females integrating into a female group associated with a nearby male [36]. It has been previously shown that dominance rank (Pearson's r = 0.527, [37]) and cortisol responsiveness (after 1 h: Pearson's r = 0.686 [37]; after 2 h: Pearson's r = 0.797 [37], R = 0.427 [29]) are stable in male guinea pigs, and that the social environment can have a profound impact on the endocrine profile of males [18, 33, 38]. However, it is not known whether cortisol concentrations are repeatable and associated with dominance rank in female guinea pigs. To address this question, we observed social interactions and calculated the dominance rank index for each of 12 individuals at two separate time points. We also carried out two cortisol response tests for each individual, in which baseline cortisol and cortisol responsiveness 1 and 2 h after the onset of a stressor were measured. We hypothesized that dominance rank and cortisol responsiveness would be repeatable and that baseline cortisol would be much less repeatable than cortisol responsiveness. Furthermore, we hypothesized that dominance rank would be correlated to cortisol measurements, although we were unsure in which direction this relationship would be. Previous studies have demonstrated that the direction of this association is based on energy demands of maintaining or enduring a certain dominance status [13, 21], and escalated aggression is generally not observed in interactions among female guinea pigs [12, 39].

## Results

**Fig. 1:**
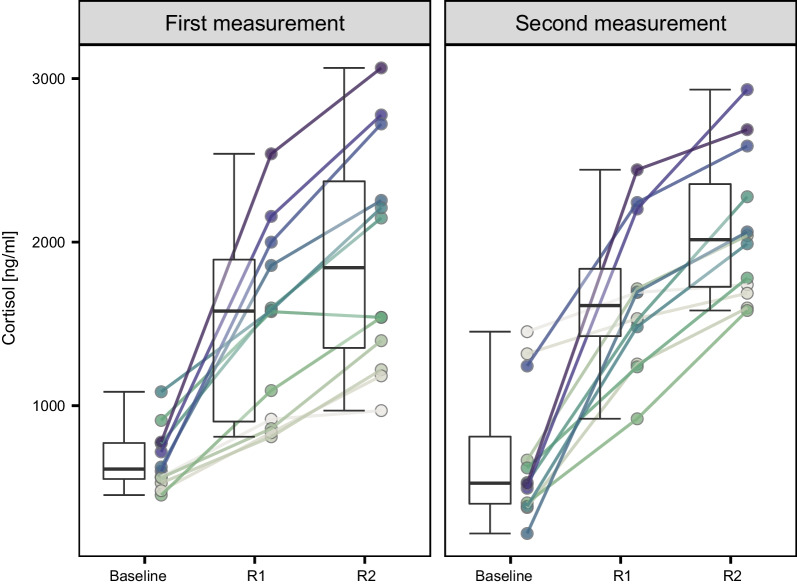
Individual values and box plots of baseline cortisol, cortisol responsiveness after 1 h (R1), and cortisol responsiveness after 2 h (R2) values at the first (left) and second (right) measurements. The values for each individual are plotted by dots connected by lines. For ease of visualization, individual is designated by color; position along the gradient is based on R2 value at first measurement

**Fig. 2:**
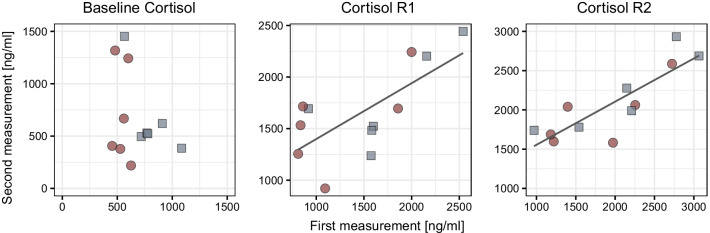
Scatterplots showing baseline cortisol (left), cortisol responsiveness after 1 h (R1; middle), and cortisol responsiveness after 2 h (R2; right) measured for each individual at the first (x axis) and second (y axis) measurement (12 individuals in each plot). The two housing groups are designated by shape and color. Baseline cortisol was not repeatable (R = 0, *p* = 0.5); cortisol responsiveness R1 and R2 were significantly repeatable (R1: R = 0.635, *p* = 0.019; R2: R = 0.764, *p* = 0.002). Lines for R1 and R2 are for visualization of the relationship and plotted from basic linear regression (second measurement ~ first measurement)

**Fig. 3:**
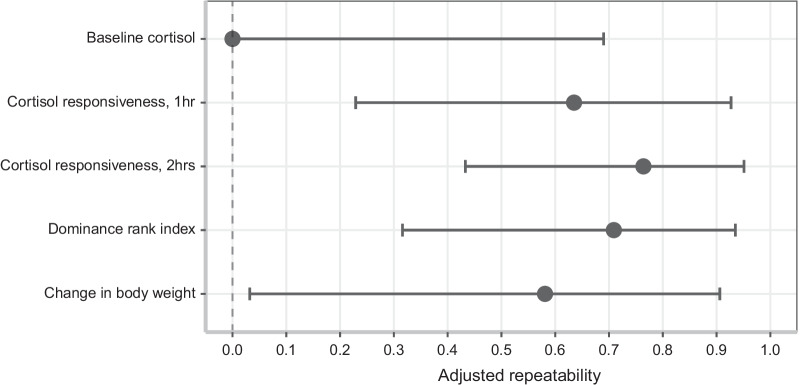
Forest plot summarizing adjusted repeatability estimates and 95% confidence intervals. Cortisol measurements (baseline and responsiveness after 1 and 2 h) were adjusted for rank index, body weight, measurement (first or second), and housing group. Rank index was adjusted for age, body weight, measurement (first or second), and housing group. Body weight lost during the cortisol response test was adjusted for age, measurement (first or second), and housing group

**Fig. 4:**
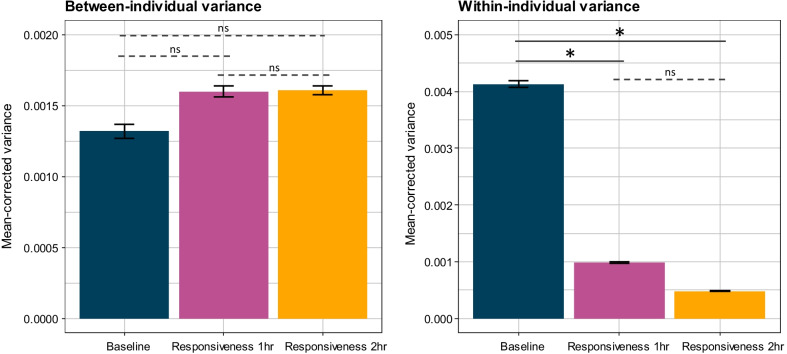
Within-individual and between-individual variance components (mean-corrected) estimated from bootstrapping (N = 1000) in the package rptR. Mean and standard error is shown. Baseline cortisol, cortisol responsiveness after 1 h, and cortisol responsiveness after 2 h did not significantly differ in the between-individual variance component. Within-individual variance was significantly higher in baseline cortisol than in cortisol responsiveness after 1 and 2 h

Overall, cortisol values significantly increased throughout the cortisol response test. This increase occurred both from the baseline level to responsiveness at 1 h (Estimated marginal means: 679, 1574; df = 10.2, t ratio = 5.777, *p* < 0.001; Fig. 1) and from responsiveness at 1 h to responsiveness at 2 h (Estimated marginal means: 1574, 2018; df = 10.0, t ratio = 5.129, *p* < 0.001; Fig. 1). Cortisol responsiveness was strikingly stable within individuals (Fig. 2), and indeed, cortisol responsiveness was significantly repeatable 1 and 2 h after exposure to a novel environment (Table 1, Fig. 3). Baseline cortisol, however, was not significantly repeatable (Table 1, Figs. 2, 3). Between-individual variance did not significantly differ between baseline cortisol and cortisol responsiveness after 1 and 2 h (baseline/responsiveness 1 h: *p* = 0.682; baseline/responsiveness 2 h: *p* = 0.688; responsiveness 1 h/responsiveness 2 h: *p* = 0.984; Fig. 4). However, the within-individual component of variance in baseline cortisol was significantly higher than the within-individual variance in cortisol responsiveness after one (*p* = 0.036; Fig. 4) and two (*p* = 0.004; Fig. 4) hours. Within-individual variance did not significantly differ between cortisol responsiveness after 1 and 2 h (*p* = 0.340; Fig. 4).

**Table 1:** Adjusted repeatability estimates for measured traits from the cortisol response test and rank index

Response variable	R (SE)	95% CI	*p*
Baseline cortisol	0 (0.217)	[0, 0.690]	0.5
Cortisol responsiveness (1 h)	0.635 (0.185)	[0.229, 0.927]	**0.019**
Cortisol responsiveness (2 h)	0.764 (0.140)	[0.433, 0.951]	**0.002**
Rank index	0.709 (0.156)	[0.316, 0.935]	**0.007**
Change in body weight (absolute)	0.581 (0.212)	[0.032, 0.906]	**0.033**

Cortisol measurements (baseline and responsiveness) are adjusted for rank index, body weight, measurement (first or second), and housing group. Rank index and change in body weight are adjusted for the fixed effects listed in Table 2. Bold indicates significant values (*p* < 0.05). N = 24 observations, 12 individuals for all models

Body weight lost during the cortisol response test was also significantly repeatable (Table 1, Fig. 3) and was not significantly correlated to cortisol responsiveness, age, or measurement. While both groups had similar body weights at the start of the cortisol response test (Group 1: mean = 703.26, standard deviation = 87.27; Group 2: mean = 691.77, standard deviation = 66.98), group 2 tended to lose more weight over the course of the test than group 1 (Group 1: mean = 5.20, standard deviation = 3.87; Group 2: mean = 9.73, standard deviation = 5.38; Table 2).

**Table 2:** Models describing variation in response variables

Response variable	Fixed effects	Estimate	SE	df	t value	P
Rank index	(Intercept)	0.700	0.086	9.123	8.128	
	Mass	0.002	0.0007	9.938	2.599	**0.027**
	Age	0.007	0.003	8.651	2.079	*0.069*
	Measurement	− 0.411	0.132	9.947	− 3.122	**0.011**
	Group	0.073	0.092	8.196	0.787	0.454
Change in body weight (absolute difference)	(Intercept)	1.689	0.362	10.140	4.663	
	Cortisol R2	0.0001	0.0003	16.160	0.298	0.769
	Age	0.014	0.013	8.777	1.147	0.282
	Measurement	− 0.624	0.552	11.100	− 1.129	0.283
	Group	0.762	0.391	8.949	1.951	*0.083*

Bold indicates significant values (*p* < 0.05); italics indicates trend (0.05 ≤ *p* < 0.1). N = 24 observations, 12 individuals for both models. The reference category for measurement is the second measurement

**Fig. 5:**
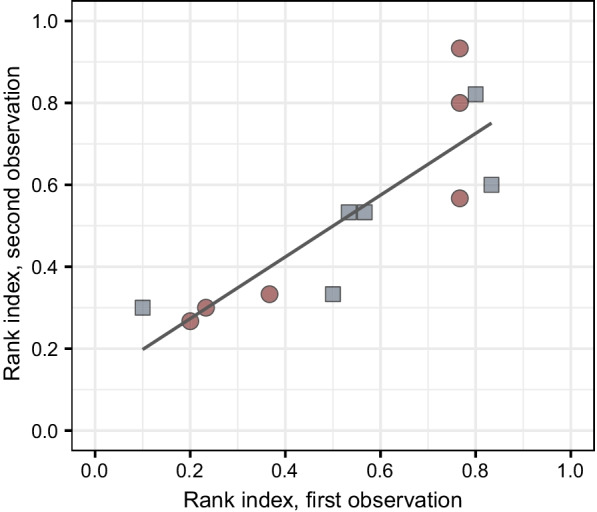
Scatterplot of rank index calculated for each individual at the first (x axis) and second (y axis) observation. The two housing groups are designated by shape and color. Rank index was significantly repeatable (R = 0.709; *p* = 0.007). Line of best fit is plotted for visualization of the relationship from basic linear regression (second observation ~ first observation)

Rank index was also significantly repeatable (Table 1, Figs. 3, 5). Mass and measurement were significantly correlated to rank index; heavier females were more dominant, and in general, higher rank order indices were observed at the second measurement (Table 2). Furthermore, older females tended to be more dominant (Table 2). Housing group did not correlate to rank index, meaning that the range of individual rank order indices observed in each group were not significantly different (Table 2, Fig. 5).

Rank index was not significantly correlated to cortisol concentrations, neither when all sampling times (baseline, responsiveness after 1 h, responsiveness after 2 h) were pooled nor when each sampling time was allowed to independently interact with rank (Table 3). Cortisol concentrations were furthermore not significantly correlated to body weight or measurement (first or second), although one housing group tended to have higher cortisol concentrations than the other housing group (Table 3).

**Table 3:** Model summary from the linear mixed effect model utilized to determine whether dominance rank index was correlated to cortisol concentrations, controlling for potential effects of body weight, habituation (measurement), and housing group

Fixed effects		Estimate	SE	df	t value	*p*
Intercept		556.109	87.590	59.815	6.349	
Sample	Responsiveness 1 h	894.135	154.773	11.149	5.777	** < 0.001**
	Responsiveness 2 h	1338.281	179.651	10.783	7.449	** < 0.001**
Rank		− 400.137	315.970	58.673	− 1.266	0.210
Sample * rank	Responsiveness 1 h	− 259.510	536.034	25.732	− 0.484	0.632
	Responsiveness 2 h	− 122.233	606.082	26.501	− 0.202	0.842
Body weight		0.451	0.951	29.957	0.474	0.639
Measurement		70.977	91.711	47.019	0.774	0.443
Housing group		175.618	97.352	27.083	1.804	*0.082*

Bold indicates significant terms (*p* < 0.05); italics indicates trend (0.05 ≤ *p* < 0.1). N = 72 observations (12 individuals, two measurements of three samples each)

## Discussion

### Repeatability of baseline cortisol, cortisol responsiveness, and body weight loss during cortisol response test

Cortisol responsiveness was significantly repeatable at both sampling times (1 and 2 h after being placed in a novel environment), but baseline cortisol was not repeatable. Despite the low sample size of the current study (N = 12 individuals), this result is consistent with recent meta-analyses [28, 30, 31] as well as current research with male guinea pigs [29, 37] that consistently find cortisol responsiveness to be more repeatable than baseline cortisol. Repeatability is calculated as the proportion of total phenotypic variance that is attributed to the between-individual component, and decomposing variance into the within- and between-individual components aids in understanding why measurements differ in repeatability [32]. Our results suggest that this difference in repeatability is due to higher within-individual variation in baseline cortisol concentration than that for cortisol responsiveness. Baseline cortisol fluctuates based on factors such as circadian rhythm and current activity patterns [14]. Circadian rhythm was controlled for by always taking the samples at the same time of day, but the activity of individuals directly before the sampling procedure was not assessed. Therefore, this high within-individual variation could be due to inconsistent behavior of individuals prior to sample collection, and we interpret baseline cortisol as an indicator of individual state. Meanwhile, the stimulus directly before cortisol responsiveness sample collection was standardized, which would decrease the within-individual variation and increase repeatability of this measurement. On the other hand, cortisol responsiveness is a strong indicator of the maximum capacity of the adrenal cortex to secrete cortisol in a variety of species including the guinea pig [40, 41] and thus cortisol responsiveness is more likely to represent a stable individual trait. Overall, these findings highlight that baseline cortisol and cortisol responsiveness are distinct measurements. Maintaining this distinction is important in studies in which it is not possible to measure stressor-induced cortisol levels or samples cannot be taken within a short timespan after disturbance to adequately measure baseline cortisol concentration.

Weight loss over the course of the cortisol response test was significantly repeatable but was not correlated to the increase in cortisol during the test. Since cortisol is a metabolic hormone, we expected individuals with the highest cortisol responsiveness to lose the most weight during the test. However, food and water were provided during the cortisol response test. Since stress-induced weight loss is often mediated by decreased appetite [42], individual differences in food uptake during the test might have led to the repeatability of weight loss rather than individual differences in cortisol responsiveness.

### Repeatability and correlates of dominance rank

The dominance rank index of females in our two social groups remained stable over the study period of six weeks. This is consistent with previous research that found that female guinea pig groups form stable (Spearman rank correlation coefficient between 0.714 and 0.976) and linear dominance hierarchies [12]. This result adds to the limited research on female dominance rank in rodents and is crucial for understanding how females cope with the social environment. Research in primate species has found that long-term rank stability can lead to differential reproductive success [3], behavior [43], or survival [44] among individuals of a group, and these consequences of female dominance merit investigation in a variety of species.

Additionally, our results show that heavier females were more dominant, and older females tended to be more dominant. Age and body weight are associated with female dominance status in many group-living mammal species [45–48]. Females with a higher body weight likely outcompete lighter females for resources, and with priority access to food, dominant individuals can maintain and potentially increase the asymmetry in body weight. However, it remains an open question whether asymmetries in weight precede dominance acquisition or emerge after dominance relationships are settled. It is important to reiterate that the groups of females in the present study were established upon weaning, thus the younger females joined the group later. It was likely difficult for the younger, newer members to become dominant over the older residents. This method of group establishment generated an age-graded group structure which is often observed in nature where juveniles integrate into established groups. This occurs in many female mammal groups [49–51], including the wild form of domestic guinea pigs, wild cavies (*Cavia aperea*) [35, 36].

### Link between dominance rank and cortisol

We did not find evidence for a relationship between dominance rank index and cortisol concentrations in this population of female guinea pigs. This is in agreement with previous studies with male guinea pigs [29, 37] which failed to detect a correlation between dominance rank and cortisol levels. The females used in the present study were housed with these groupmates for several months prior to the beginning of the study, and indeed we found that rank index was repeatable. Therefore, we assume that living in a stable social group is not particularly stressful for females of different ranks.

It is important to frame this lack of correlation between dominance rank and cortisol concentrations within the limitations of the present study. We studied two social groups, each consisting of six individuals. Number of group members can influence social structure and attributes of the dominance hierarchy. Schuhr [10] observed social behavior in different group sizes of female mice and found that the social structure and social behavior of high and low ranking individuals differed based on the group size. Thus, the group size and number of groups observed can have a profound influence on the social structure and whether other measurements correlate to rank. Additionally, the animals in this study were fed ad libitum, and harsher environmental conditions might have resulted in a stronger relationship between dominance rank index and cortisol concentrations [13]. Finally, it is highly recommended to include repeated measures when investigating correlations between labile traits such as hormones and behavior to account for within-individual variation [23]. We measured each trait twice for each individual, but we acknowledge that increasing the number of repeated measurements per individual and/or the number of individuals included in the study would strengthen the interpretation of our results. Taken together, further research addressing these limitations is needed to fully understand whether there is a link between dominance rank and cortisol concentrations in this study system.

## Methods

### Animals and husbandry

The guinea pigs used in this experiment were bred at the Department of Behavioural Biology at the University of Münster. The breeding program was established in 1975 with 40 founder multicolored shorthair guinea pigs from a professional breeder, and individuals from other breeders are routinely added to the breeding stock to prevent inbreeding. The breeding program consists of multiple breeding harems, whereby one male and two to three females are housed together in an enclosure with an area of either 1m^2^ or 1.5m^2^. The offspring of these individuals remain in the harem groups throughout weaning and are removed at 21 ± 1 days of age.

The twelve animals used in this study were transferred upon weaning to two groups of six females. Since the females were born at slightly different times, the groups were established when the oldest two females were at least 20 days old, and the younger females were added as they reached 21 days of age. The age difference between the oldest and youngest female of each group was 59 and 57 days. This age variation is random due to the continuous breeding program. No full siblings were used for this experiment; any half-siblings shared a father and were subsequently housed in separate groups.

The area of each enclosure was 1.5m^2^; the walls had a height of 0.5 m and were made of wood with a red plastic segment at the bottom, and the floor was covered with wood shavings (Tierwohl Super, J. Rettenmaier & Söhne GmbH + Co KG, Rosenberg, Germany). Each enclosure contained two large shelters made of red plastic, hay that was refreshed daily, and commercial guinea pig food (Höveler Meerschweinchenfutter 10,700, Höveler Spezialfutterwerke GmbH & Co. KG) and water were supplied ad libitum; water was supplemented weekly with vitamin C powder. The two groups were housed in separate rooms under controlled conditions, with a light dark cycle of 12:12 (lights on 7:00–19:00), average temperature of 22 °C, and average humidity of 42%.

### Experimental procedure

**Fig. 6:**
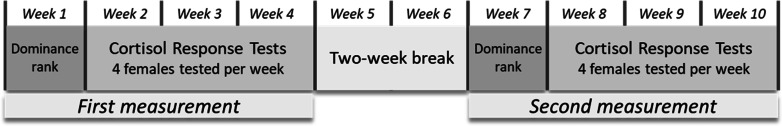
Experimental procedure of rank index determination and cortisol response tests. Videos analyzed for rank index were recorded in weeks 1 and 7. Weeks 2 through 4 and 8 through 10 consisted of cortisol response tests; four females were tested per week. The same testing order was maintained for the second measurement; for example, the same four females that underwent the cortisol response test in week 2 had their second cortisol response test in week 8

Testing occurred in two four-week testing phases (Fig. 6). In the first week of each testing phase, videos were recorded in the home enclosures to be later analyzed to determine dominance rank indices of individuals. In the following three weeks, a cortisol response test was carried out for each female. Two females from each group were tested each week and only one female from each group was tested each day. The second testing phase began after a two week break so that there were six weeks between each measurement, and the sequence of individuals tested in weeks 8 through 10 was identical to the order of individuals tested in weeks 2 through 4. Mean age at the first cortisol response test was 172 days (range 151–197 days); mean age at the second cortisol response test was 213 days (range 193–238 days). Females had lived in their social groups for at least four months prior to the first observation.

### Rank index determination

Rank index was determined from videos recording the social behavior of the groups over the course of a week. Videos were recorded for multiple hours in the afternoon every other day (Monday, Wednesday, and Friday), and this was repeated after a six week break to assess the stability of rank index. The behavioral coding software *Interact* (Interact, Lab Suite Version 2017, Mangold International GmbH) was used for video analysis. Individuals were observed using focal animal sampling, and each individual was observed until it was involved in 10 interactions resulting in a retreat for each of the three days. Therefore, each female had a total of 30 interactions in which she retreated from another female or another female retreated from the focal female. A retreat was defined as the following: A female moves away from another female so that she maintains a distance of more than one body length; this behavior is shown either after an interaction of the females or after an approach of one of the females involved. Female guinea pigs do not often interact agonistically and can be quite subtle in their dominance interactions [12, 39]. Therefore, this retreat definition allowed for responses to interactions as well as responses to approaches, as long as the females had been within one body length of one another. A retreat was counted even if other females were nearby as long as the two interacting females were within one body length of one another. A rank order index was calculated for each female as the ratio of the number of times the focal female was retreated from divided by the total number of retreats (30). Therefore, the rank order index was on a scale from 0 to 1, with 0 being completely subdominant and 1 being completely dominant. This rank order index was calculated separately for the first and second measurement for each individual.

### Cortisol response test

The cortisol response test is used to measure the endocrine stress response to a challenge. Cortisol is the predominant glucocorticoid in guinea pigs [52], and guinea pigs show an increase in plasma cortisol when exposed to a novel environment void of shelter [53]. It is not known how long cortisol concentrations increase in response to a challenging situation before peaking and decreasing in female guinea pigs. For this experiment, individual females were placed in a novel enclosure for 2 h, and blood samples were taken directly before and after 1 and 2 h to capture the baseline and response values of plasma cortisol.

The cortisol response test began at 13:00 ± 15 min, as plasma cortisol concentrations fluctuate throughout the day and a peak is observed at 13:00 [40]. By starting the cortisol response test at the daily cortisol peak, any increase in cortisol concentration measured would be due to the challenge and not circadian fluctuations. The dimensions of the test enclosure were 1 m × 1 m, with walls that were 50 cm high constructed of wood with a red plastic section at the bottom. Similar to housing conditions, the floor of the enclosure was covered with wood shavings and water and guinea pig food was provided. The test enclosure was in a different guinea pig housing room from where the focal individual was housed.

The housing room of the focal individual was locked 1 h prior to the beginning of the test to prevent any influence of human activity on baseline cortisol levels. At the beginning of the cortisol response test, a stopwatch was started directly when the experimenter knocked on the door to enter the housing room of the focal animal. The focal animal was collected, brought to a separate room, and placed on the lap of an assistant who then applied a small amount of muscle salve (Finalgon® salve, Boehringer Ingelheim International GmbH, Ingelheim on the Rhine, Germany) to the ears of the focal animal; excess salve was removed directly afterward. The assistant then held the ear of the animal taut so that the experimenter could illuminate the blood vessels via a cold-point lamp held beneath the ear and prick a visible blood vessel with a sterile blood lancet (Solofix® Blutlanzetten, B. Braun Melsungen AG, Melsungen, Germany). The assistant massaged the ear to stimulate blood flow while the experimenter collected approximately 150 μl of blood with two capillary tubes (Capillary tubes for microhaematocrits, 100 μl, Paul Marienfeld GmbH & Co KG, Lauda-Königshofen, Germany) within three minutes of entering the housing room of the focal animal. A swab was applied to the ear to stop the blood flow, and the focal animal was weighed (to the decigram) and placed into the test enclosure with the rump against the center of the closest wall. The room was then locked to prevent disturbance during the test, and the blood sample and weighing procedure was repeated 60 min and 120 min after the initial entering of the focal animal housing room.

The blood plasma was isolated directly after the blood sampling procedure. The capillary tubes were sealed at one end with hematocrit sealing compound (Brand GmbH & Co. KG, Wertheim, Germany). The sealed capillary tubes were then centrifuged at 13,000 rpm (16,060 g) for five minutes, after which the plasma was separated from the rest of the blood. The capillary tube was broken at this separation point with an electronic file, and the plasma was pipetted into an Eppendorf tube and centrifuged at 13,000 rpm (13,800 g) for three minutes. The plasma was pipetted into a new Eppendorf tube and centrifuged under the same conditions until no visible pellet remained. The plasma samples were then frozen at − 20 °C.

The concentration of cortisol in the blood plasma was determined using an enzyme-linked immunosorbent assay (Cortisol ELISA, RE52061, IBL International GmbH, Hamburg, Germany). The samples were analyzed in two batches consisting of six individuals each. The principle of the analysis is based on the following description (IBL International GmbH 2014):

A certain amount of enzyme-labelled antigen and the antigen in the sample compete for the binding sites of the antibody-coated wells. After a certain incubation time, the enzyme-labelled antigens that had not bound were removed by washing. The substances prednisolone (30%), 11-desoxy-cortisol (7%), corticosterone (1.4%), cortisone (4.2%), prednisone (2.5%), 17α-oh-progesterone (0.4%), desoxy-corticosterone (0.9%) and 6α-methyl-17α-oh-progesterone cross-reacted with the antibody. The intra-assay variances were on average CV = 2.98% and the inter-assay variances were on average CV = 3.51%.

### Statistical analysis

Statistical analysis was carried out with R version 4.0.3 [54]. The package rptR (version 0.9.22) [55] was used to estimate adjusted repeatability for baseline cortisol, cortisol responsiveness, rank index, and body weight lost during the cortisol response test. In addition, the packages lme4 (version 1.1.25) [56] and lmerTest (version 3.1.3) [57] were used to assess the influence of the fixed effects on rank index and body weight lost during the cortisol response test. Performance (version 0.7.3) [58] was used to verify that the models fulfilled assumptions. When using rptR, permutation was set to 500 and bootstrapping was set to 1000. Two-tailed tests were used and the significance threshold was set at 0.05.

Linear mixed-effect models were fitted to estimate adjusted repeatability of baseline cortisol (log transformed), cortisol responsiveness after 1 h (log transformed), cortisol responsiveness after 2 h (log transformed), rank index, and body weight lost during the cortisol response test (absolute values log transformed). Continuous fixed effects were mean-centered and individual identity was fitted as a random effect in all models. To control for any influence of time or habituation to the testing regime or any influence of the housing group, measurement (first or second) and housing group were included as fixed effects in all models. Baseline cortisol and cortisol responsiveness after 1 and 2 h included rank index and body weight as additional fixed effects. Age and body weight were included as fixed effects for rank index. For body weight lost during the cortisol response test, cortisol responsiveness after 2 h and age were additionally included as fixed effects.

After running the repeatability analysis for the three cortisol sampling times (baseline, responsiveness after 1 h, responsiveness after 2 h), further analyses were carried out to gain a better understanding of why cortisol responsiveness after 1 and 2 h were repeatable but baseline cortisol concentration was not. To do this, the variance was partitioned into within- and between-individual components for each cortisol sampling time. These variance components were then pairwise compared between the three cortisol sampling times. An asymptotic two-tailed P value was calculated as twice the proportion of samples in which the difference (within-individual variance component from baseline cortisol minus the within-individual variance component from responsiveness after 1 h, etc.) was smaller/greater than zero.

To assess whether there was an association between cortisol concentrations and rank index, a linear mixed-effect model was fit with cortisol concentration (untransformed) as the response variable and sampling time (baseline, responsiveness 1 h, responsiveness 2 h), rank index, and the interaction between sampling time and rank index as fixed effects. Sampling time was additionally included as a random slope, and individual identity was included as a random intercept. Covariation of slopes and intercepts was not constrained. Additional fixed effects included body weight (mean centered), measurement (first or second), and housing group. To determine whether the cortisol concentrations measured at the three sampling times (baseline, responsiveness after 1 h, responsiveness after 2 h) significantly differed, the package emmeans (version 1.6.2-1) [59] was used.

## Supplementary Information

**Additional file 1.** Text file containing the dataset analyzed for this publication.

**Additional file 2.** R script used for data analysis.

## Data Availability

All data analyzed during this study are included in the supplementary information files.
